# Supplementary material for: Exploring the roles of RNAs in chromatin architecture using deep learning
Source: Nat Commun. 2024 Jul 29;15:6373. doi: 10.1038/s41467-024-50573-w (PMC11286850; doi:10.1038/s41467-024-50573-w)
Supplement: Supplementary file 1 — Supplementary Information [file 41467_2024_50573_MOESM1_ESM.pdf]

Supplemental Information for

Exploring the Roles of RNAs in Chromatin Architecture Using Deep Learning

Shuzhen Kuang[1], Katherine S. Pollard[1,2,3*]

[1]Gladstone Institute of Data Science and Biotechnology, San Francisco, CA, USA

[2]Department of Epidemiology & Biostatistics, University of California, San Francisco, CA, USA

[3]Chan Zuckerberg Biohub, San Francisco, CA, USA

* Corresponding author. Email: katherine.pollard@gladstone.ucsf.edu

Supplementary Table 1. Data source used to extract features for AkitaR models.

Model Input	Experiment	Accession Number
Steady State RNA	RNA-seq	4DNESFH3EHTU
Nascent RNA	iMARGI	4DNES9Y1GHK4
Trans-located RNA	iMARGI	4DNES9Y1GHK4
ATAC-seq	ATAC-seq	4DNESMBA9T3L

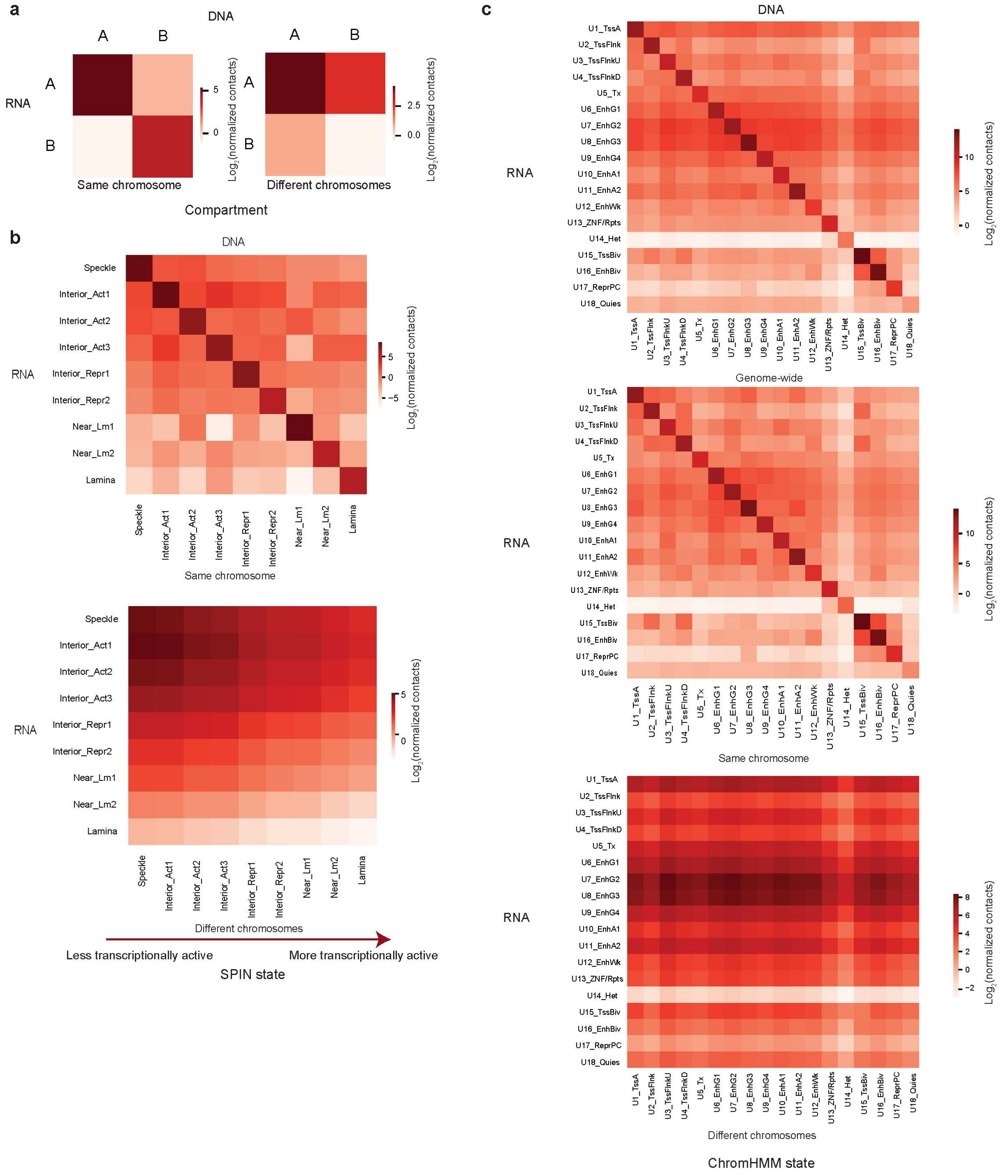

Supplementary Figure 1. RNA-DNA interactions occur across compartments, SPIN states and chromHMM states. The number of RNA-DNA interactions (log_2 normalized count) within and across compartments (a), SPIN states (b) and chromHMM states (c). RNA-DNA interactions are separated into the ones on the same chromosome and the ones on different chromosomes. For chromHMM states, genome-wide RNA-DNA interactions combining both the ones on the

same and different chromosomes are also shown. Interaction frequencies are normalized to the size of compartments, SPIN states and chromHMM states. SPIN: Spatial Position Inference of the Nuclear genome, Interior_Act 1: Interior Active 1, Interior_Act 2: Interior Active 2, Interior_Act 3: Interior Active 3, Interior_Repr1: Interior Repressive 1, Interior_Repr2: Interior Repressive 2, Near_Lm1: Near Lamina 1, Near_Lm2: Near Lamina 2, U1_TssA: Active TSS, U2_TssFlnk: Flanking TSS, U3_TssFlnkU: Flanking TSS Upstream, U4_TssFlnkD: Flanking TSS Downstream, U5_Tx: Transcription, U6_EnhG1: Genic Enhancer 1, U7_EnhG2: Genic Enhancer 2, U8_EnhG3: Genic Enhancer 3, U9_EnhG4: Genic Enhancer 4, U10_EhnA1: Active Enhancer 1, U11_EnhA2: Active Enhancer 2, U12_EnhWk: Weak Enhancer, U13_ZNF/Rpts: ZNF Genes & Repeats, U14_Het: Heterochromatin, U15_TssBiv: Bivalent/Poised TSS, U16_EnhBiv: Bivalent Enhancer, U17_ReprPC: Repressed PolyComb, U18_Quies: Quiescent. Source data are provided as a Source Data file.

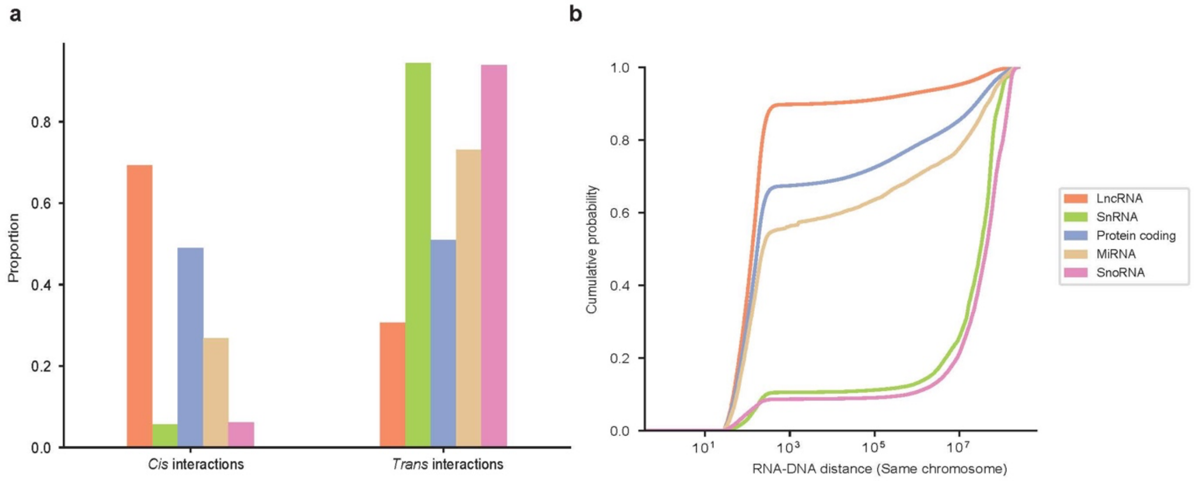

Supplementary Figure 2. RNA-chromatin interactions prevalently occur *in trans*. (a) The proportion of RNAs involved in *trans*-interactions for each RNA type. (b) The cumulative probability of the RNA-DNA interactions as a function of genomic distance between DNA and RNA loci on the same chromosome. Source data are provided as a Source Data file.

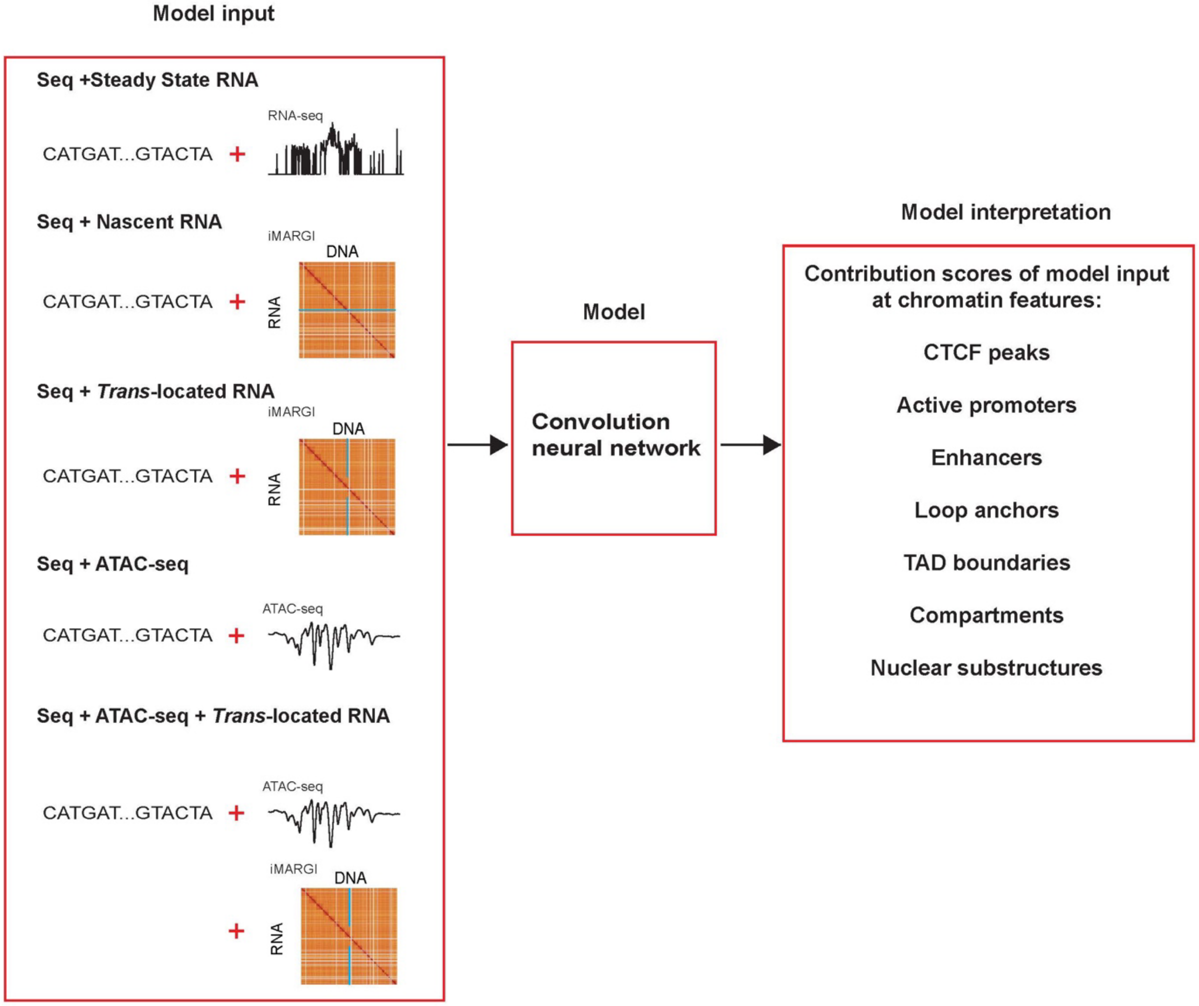

Supplementary Figure 3. Flowchart of workflow for exploring the roles of caRNAs in 3D genome architecture using deep learning. The inputs for the several models trained in this study are shown in the left panel. The chromatin features used to annotate the DNA regions with high contribution scores of different model inputs are shown in the right panel.

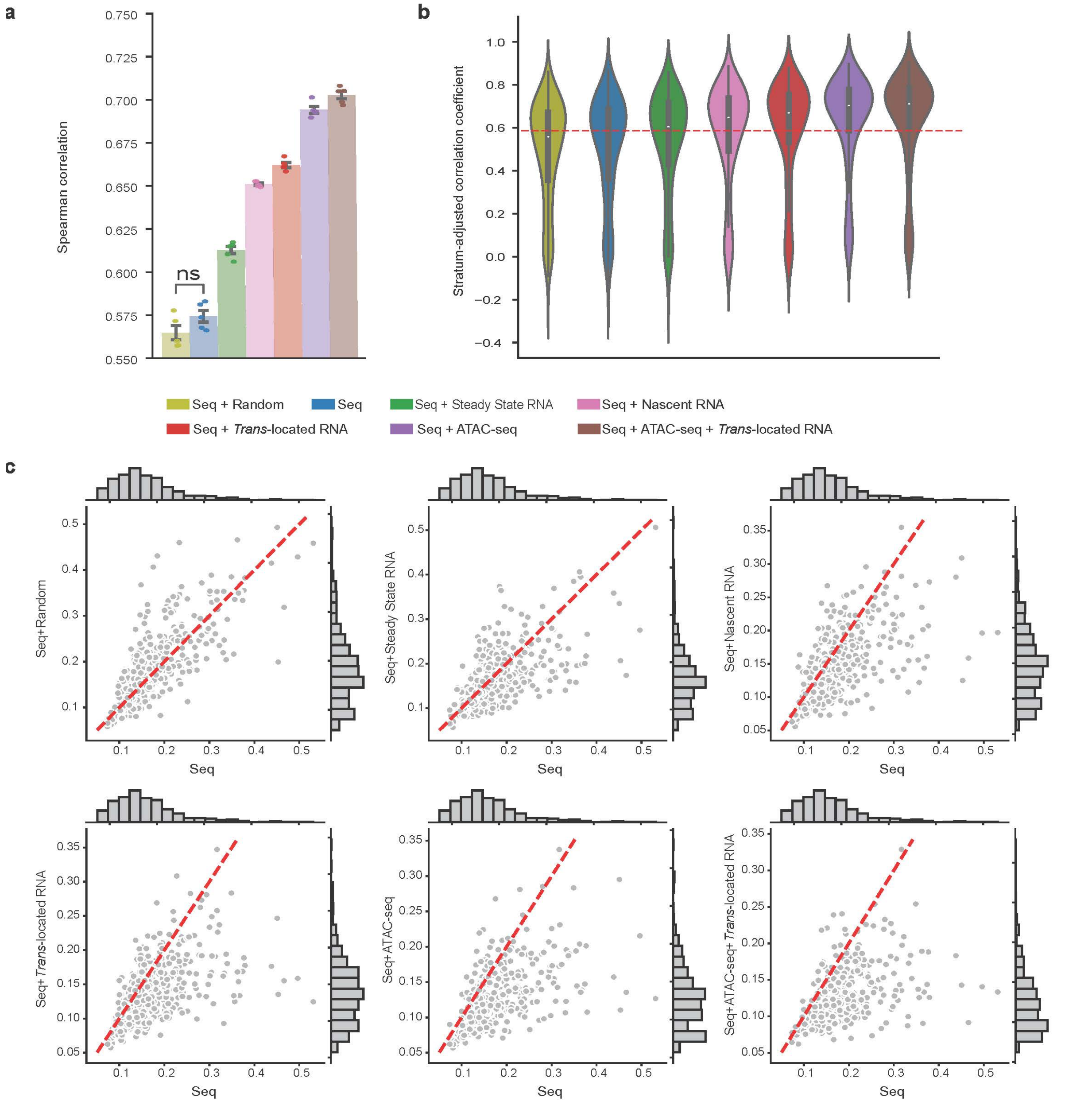

Supplementary Figure 4. Chromatin-associated RNAs increase the accuracy of contact map predictions. (a) Barplots of Spearman's correlation between experimental and predicted contact maps of the held-out test set. Error bars in the barplots represent the mean ± standard error of the mean for each model type independently trained five times. Two-sided Mann-Whitney U tests were used to evaluate differences between all pairs of models. Every comparison was significant (p-value<=0.05) except those labeled as not significant (ns). U statistics and p-values for the comparisons are shown in Supplementary Data 1. Individual data points are shown as dots. (b) Violin plot of stratum-adjusted Pearson's correlation between observed test set maps versus predicted maps (n=413) for the best model of each type. The box represents the interquartile ranges (IQR), with whiskers setting to 1.5 times the IQR. The dot within the box

represents the median value. (c) Pairwise comparisons of mean squared error (MSE) between the models with additional features and sequence alone model, based on the best model of each type. MSE was calculated per held-out test region between experimental and predicted contact maps. The points under the red line (y=x) represent the regions with lower MSE (better performance) for the model with additional features. Source data are provided as a Source Data file.

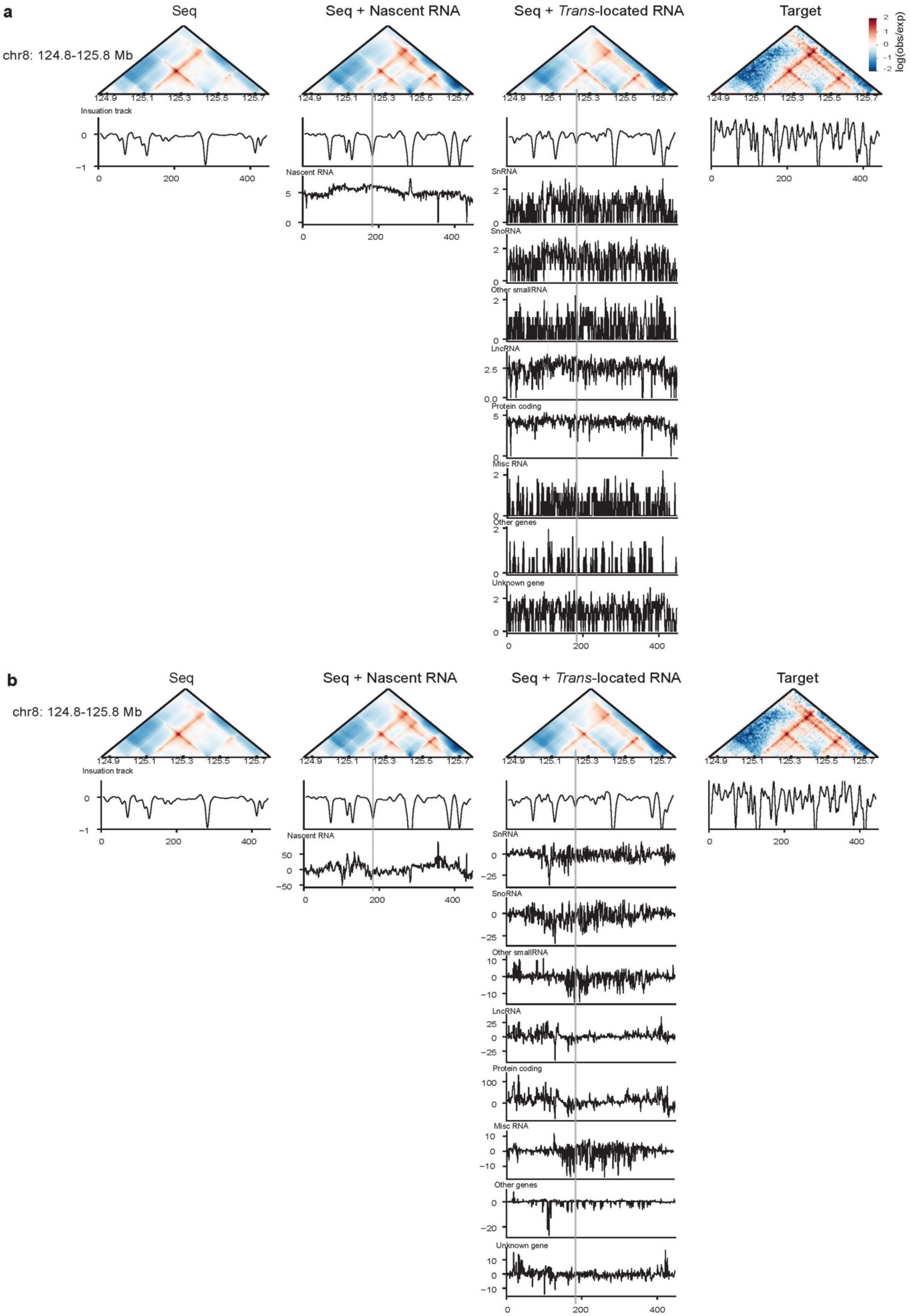

a
Seq
Seq + Nascent RNA
Seq + Trans-located RNA
Target
chr8: 124.8-125.8 Mb
log(obs/exp)
Insuation track
Nascent RNA
SnRNA
SnoRNA
Other smallRNA
LncRNA
Protein coding
Misc RNA
Other genes
Unknown gene
b
Seq
Seq + Nascent RNA
Seq + Trans-located RNA
Target
chr8: 124.8-125.8 Mb
Insuation track
Nascent RNA
SnRNA
SnoRNA
Other smallRNA
LncRNA
Protein coding
Misc RNA
Other genes
Unknown gene

Supplementary Figure 5. An example showing the better predictions of the model with nascent transcription compared to the one with *trans*-located caRNAs. Model inputs and their contribution scores are shown in a and b, respectively. Insulation tracks of the predicted or experimental contact maps were also shown. The region with better predictions by the model with nascent transcription is highlighted with a gray bar. Source data are provided as a Source Data file.

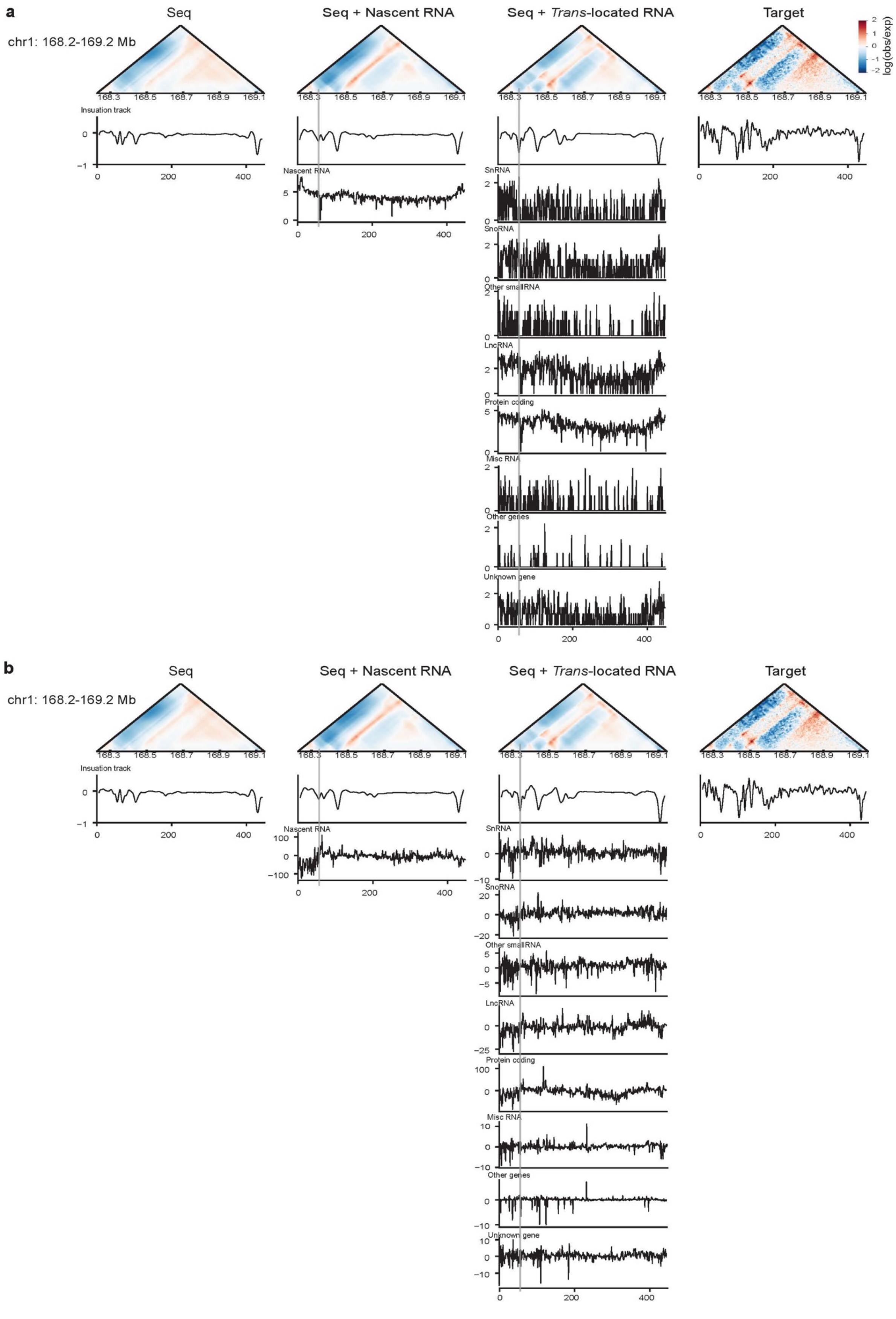

a
Seq
Seq + Nascent RNA
Seq + Trans-located RNA
Target
chr1: 168.2-169.2 Mb
log(obs/exp)
Insuation track
Nascent RNA
SnRNA
SnoRNA
Other smallRNA
LncRNA
Protein coding
Misc RNA
Other genes
Unknown gene
b
Seq
Seq + Nascent RNA
Seq + Trans-located RNA
Target
chr1: 168.2-169.2 Mb
Insuation track
Nascent RNA
SnRNA
SnoRNA
Other smallRNA
LncRNA
Protein coding
Misc RNA
Other genes
Unknown gene

Supplementary Figure 6. An example showing the better predictions of the model with *trans*-located caRNAs compared to the one with nascent transcription. Model inputs and their contribution scores are shown in a and b, respectively. Insulation tracks of the predicted or experimental contact maps are also shown. The region with better predictions by the model with *trans*-located caRNAs is highlighted with a gray bar. Source data are provided as a Source Data file.

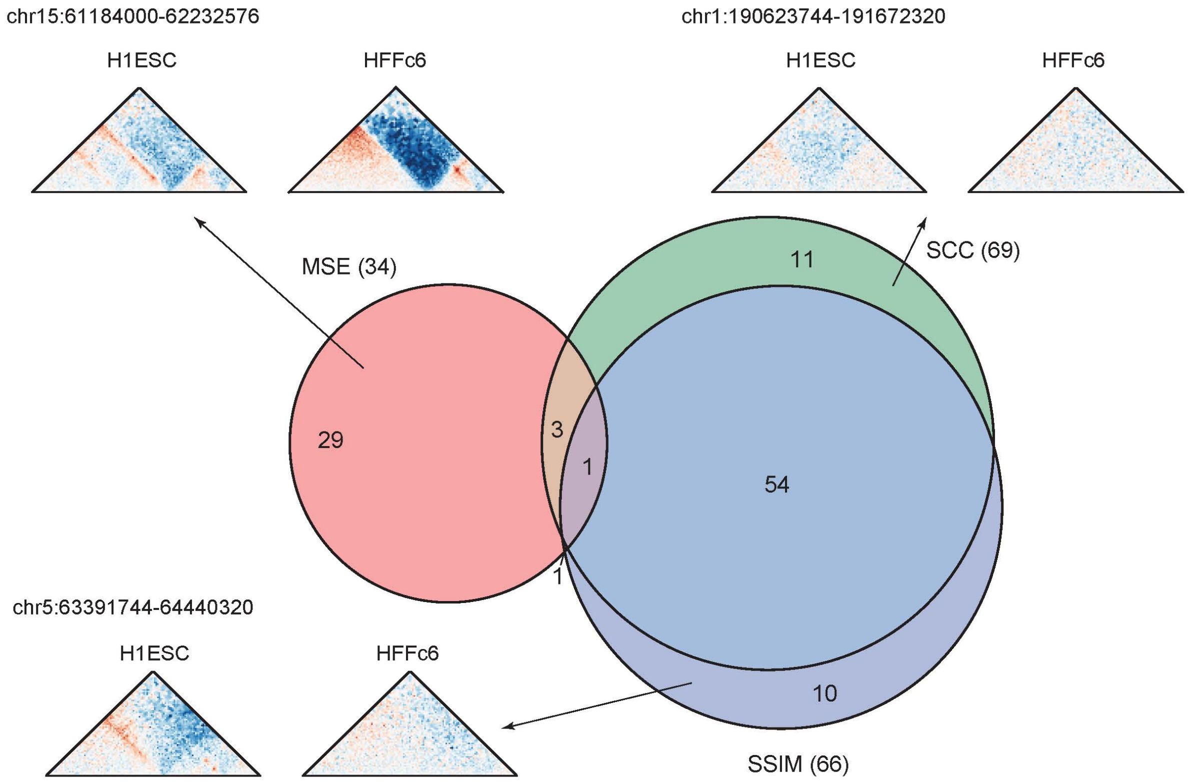

Supplementary Figure 7. Test regions with cell-type differences between Micro-C data in H1ESC versus HFFc6. The cell-type-specific regions were identified by mean squared error (MSE>0.3), stratum-adjusted correlation coefficient (SCC<0.2) or structural similarity index measure (SSIM<0.08). A representative example for the cell-type-specific regions identified by each metric is shown. Source data are provided as a Source Data file.

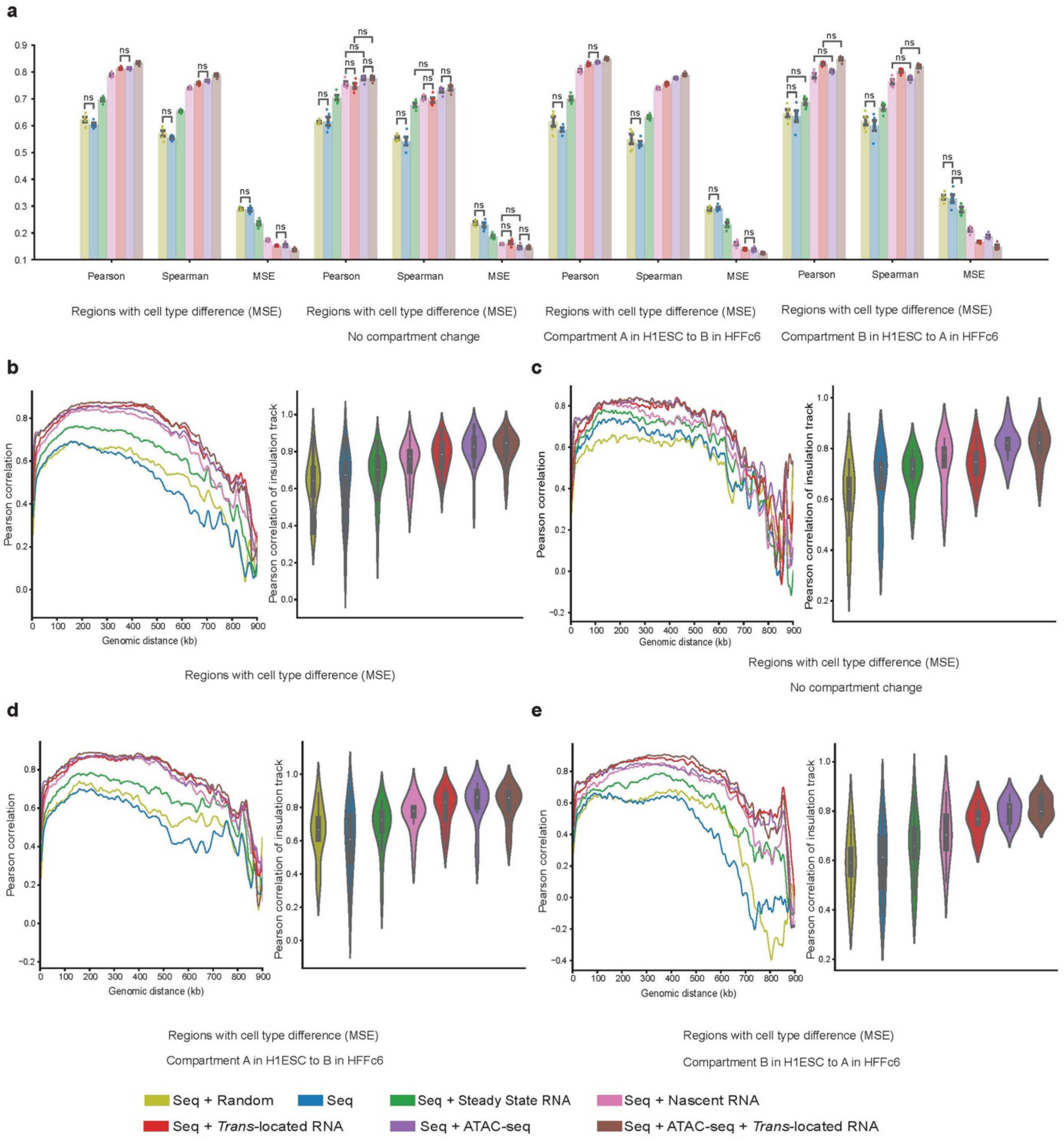

Supplementary Figure 8. Chromatin-associated RNAs help capture cell-type-specific genome folding. (a) Barplots of Pearson's correlation, Spearman's correlation and mean squared error (MSE) between experimental and predicted contact maps on the cell-type-specific subsets (MSE>0.3 between experimental maps in HFFc6 versus H1ESC) and cell-type-specific subsets (MSE>0.3) without compartment change, with compartment changes from A compartment in H1ESC to B compartment in HFFc6, or with compartment changes from B compartment in H1ESC to A compartment in HFFc6. Error bars in the barplots represent the mean ± standard error of the mean for each model type independently trained five times. Two-sided Mann-Whitney U tests were used to evaluate differences between all pairs of models. Every

comparison was significant (p-value<=0.05) except those labeled as not significant (ns). U statistics and p-values for the comparisons are shown in Supplementary Data 1. Individual data points are shown as dots. (b-e) Stratified Pearson’s correlation and violin plot of Pearson's correlation of insulation tracks between experimental and predicted contact maps on the cell-type-specific test subsets identified by MSE (MSE>0.3, n=34) (b) and cell-type-specific subsets (MSE>0.3) without compartment change (n=8) (c), with compartment changes from A compartment in H1ESC to B compartment in HFFc6 (n=17) (d), or with compartment changes from B compartment in H1ESC to A compartment in HFFc6 (n=9) (e). The best model of each type (out of five) was used. The box represents the interquartile ranges (IQR), with whiskers setting to 1.5 times the IQR. The dot within the box represents the median value. Source data are provided as a Source Data file.

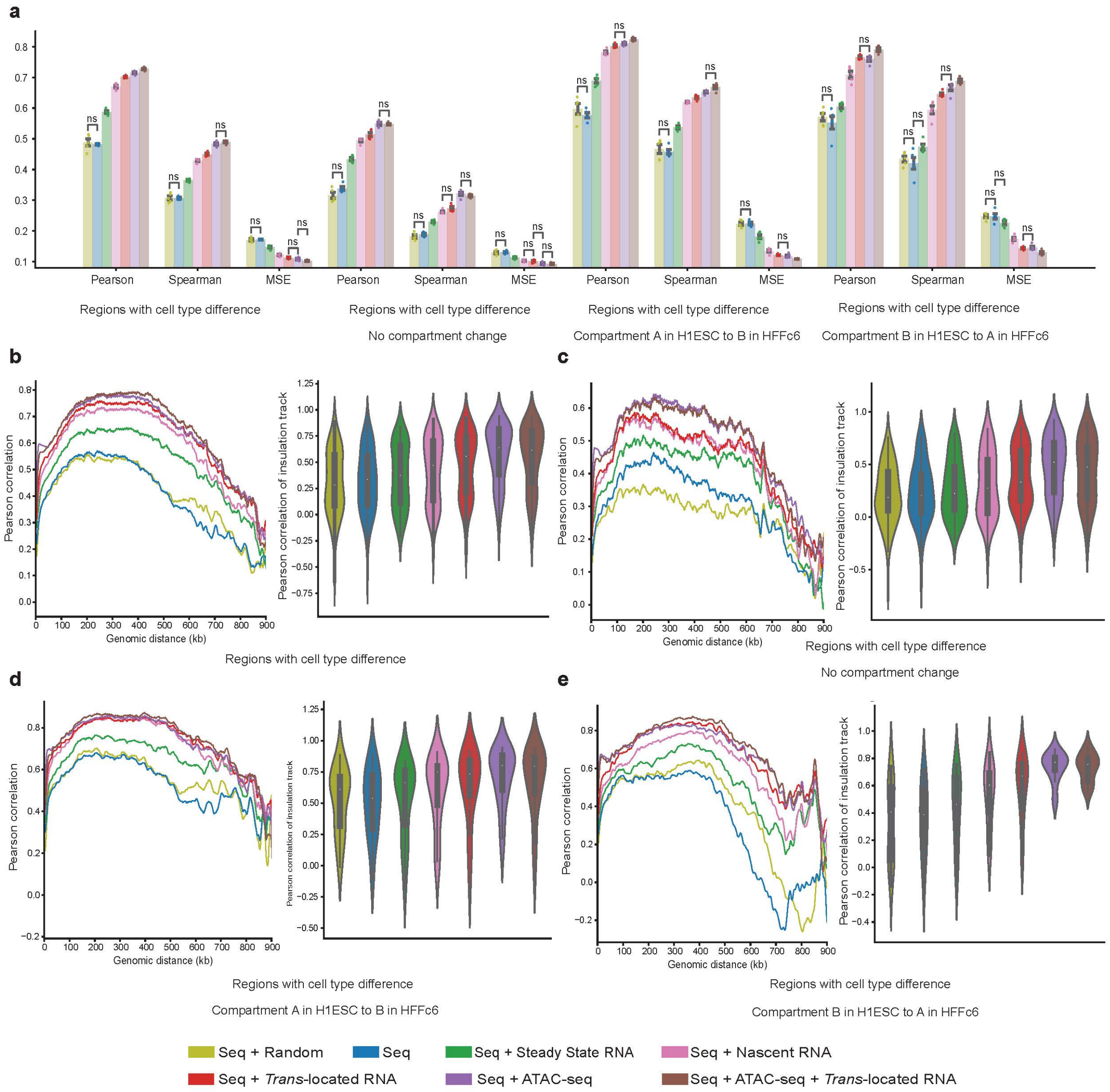

Supplementary Figure 9. Chromatin-associated RNAs help capture cell-type-specific genome folding identified by mean squared error (MSE), stratum-adjusted correlation coefficient (SCC) or structural similarity index measure (SSIM). (a) Barplots of Pearson's correlation, Spearman's correlation and MSE between experimental and predicted contact maps on the cell-type-specific subsets (MSE>0.3, SCC<0.2 or SSIM<0.08) and cell-type-specific subsets (MSE>0.3, SCC<0.2 or SSIM<0.08) without compartment change, with compartment changes from A compartment in H1ESC to B compartment in HFFc6, or with compartment changes from B compartment in H1ESC to A compartment in HFFc6. Error bars in the barplots represent the mean ± standard error of the mean for each model type independently trained five times. Two-sided Mann-Whitney U tests were used to evaluate differences between all pairs of models.

Every comparison was significant (p-value<=0.05) except those labeled as not significant (ns). U statistics and p-values for the comparisons are shown in Supplementary Data 1. Individual data points are shown as dots. (b-e) Stratified Pearson’s correlation and violin plot of Pearson's correlation of insulation tracks between experimental and predicted contact maps on the cell-type-specific test subsets (MSE>0.3, SCC<0.2 or SSIM<0.08, n=109) (b) and cell-type-specific subsets (MSE>0.3, SCC<0.2 or SSIM<0.08) without compartment change (n=65) (c), with compartment changes from A compartment in H1ESC to B compartment in HFFc6 (n=27) (d), or with compartment changes from B compartment in H1ESC to A compartment in HFFc6 (n=17) (e). The best model of each type (out of five) was used. The box represents the interquartile ranges (IQR), with whiskers setting to 1.5 times the IQR. The dot within the box represents the median value. Source data are provided as a Source Data file.

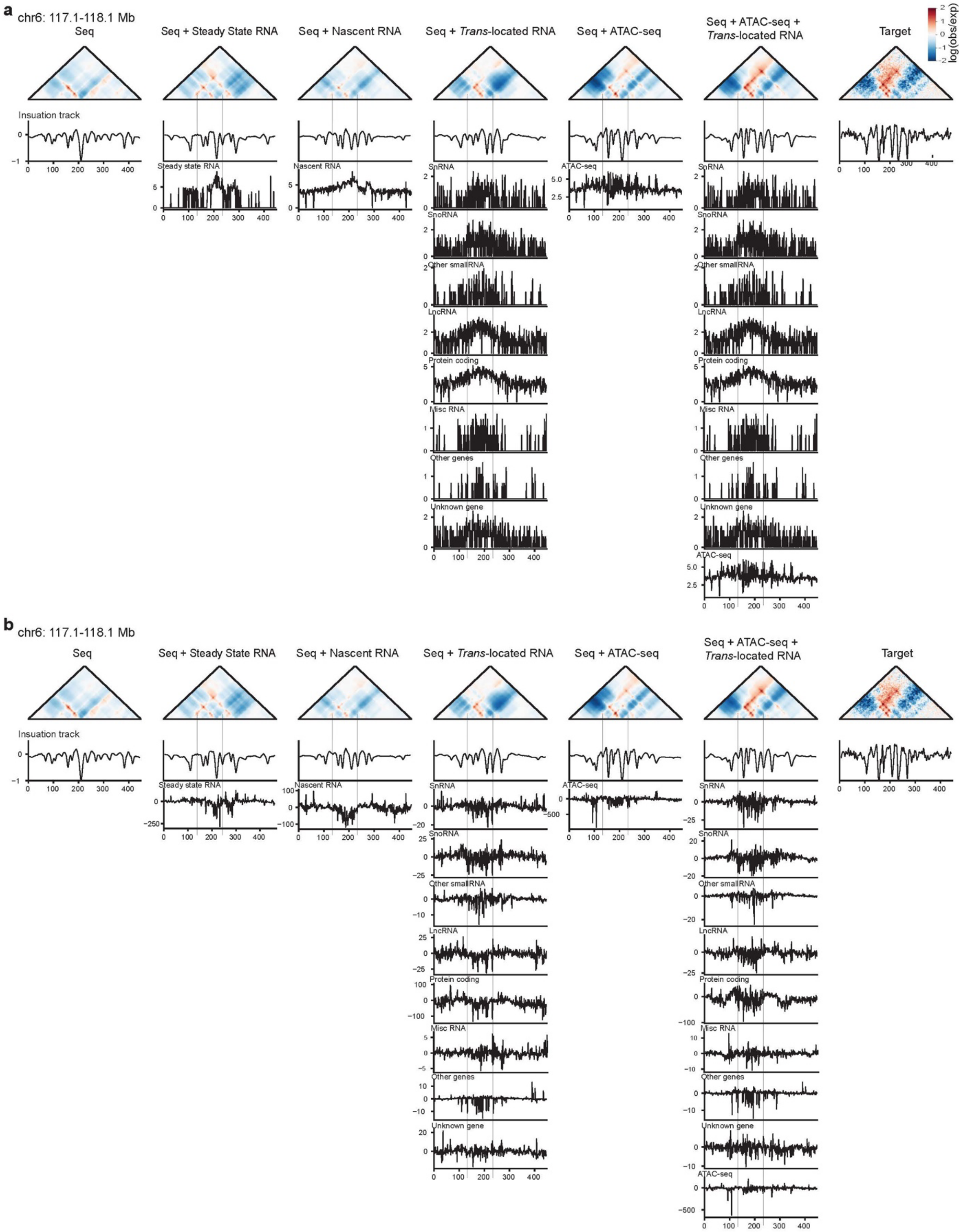

Supplementary Figure 10. An example showing the model with *trans*-located caRNAs captured some cell-type-specific chromatin interactions better than all other RNA and ATAC-seq

features. Model inputs and their contribution scores are shown in a and b, respectively. Insulation tracks of the predicted or experimental contact maps are also shown. The region with better predictions by the model with *trans*-located caRNAs is highlighted with a gray bar. Source data are provided as a Source Data file.

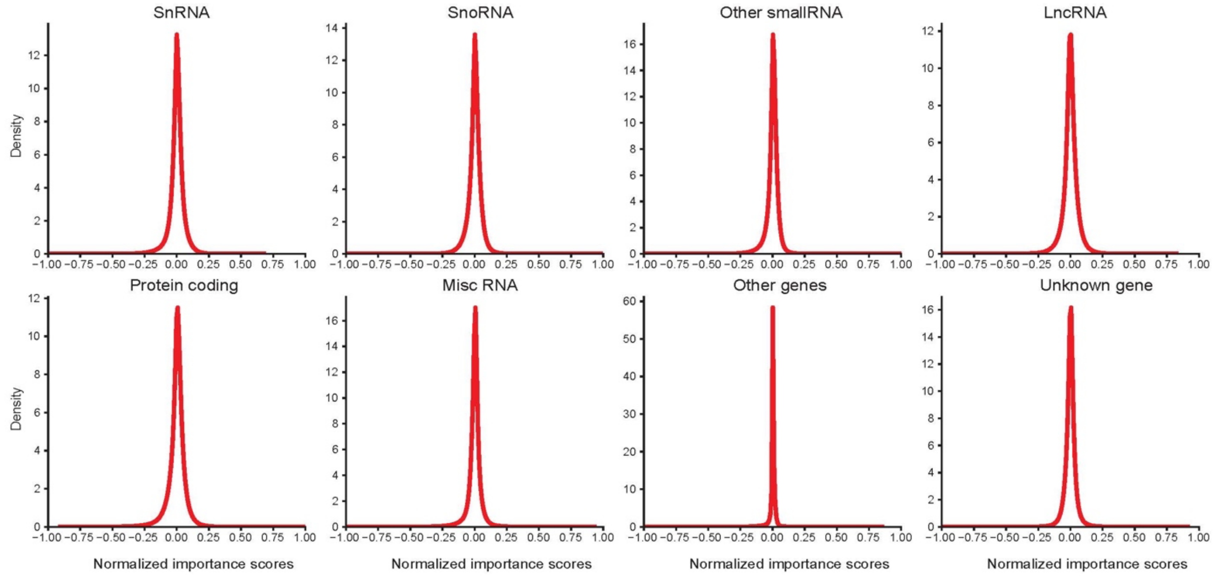

Supplementary Figure 11. Density plot of normalized contribution scores of *trans*-located caRNAs from different RNA types. Contribution scores were normalized to their maximum absolute values. SnRNAs, lncRNAs, RNAs from other types of genes and RNAs from regions without known annotation demonstrated asymmetric distribution with slightly elongated left tails. Source data are provided as a Source Data file.

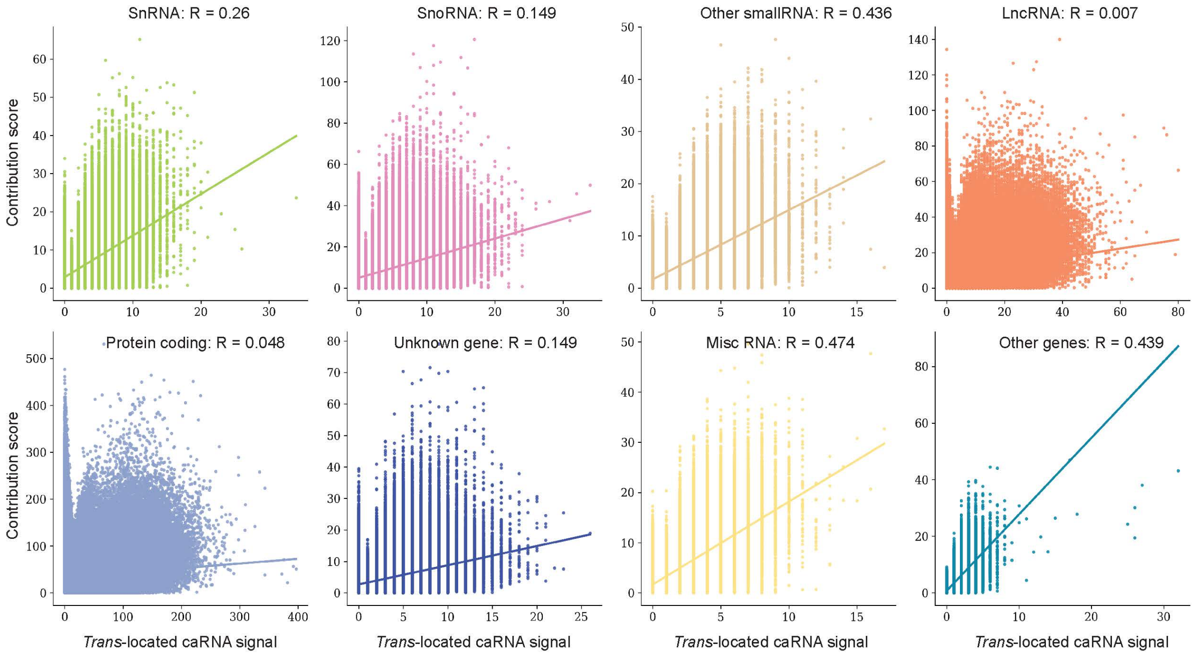

Supplementary Figure 12. Absolute contribution scores of *trans*-located caRNAs from different RNA types show low or medium correlation with their input signals (before taking the natural log). Source data are provided as a Source Data file.

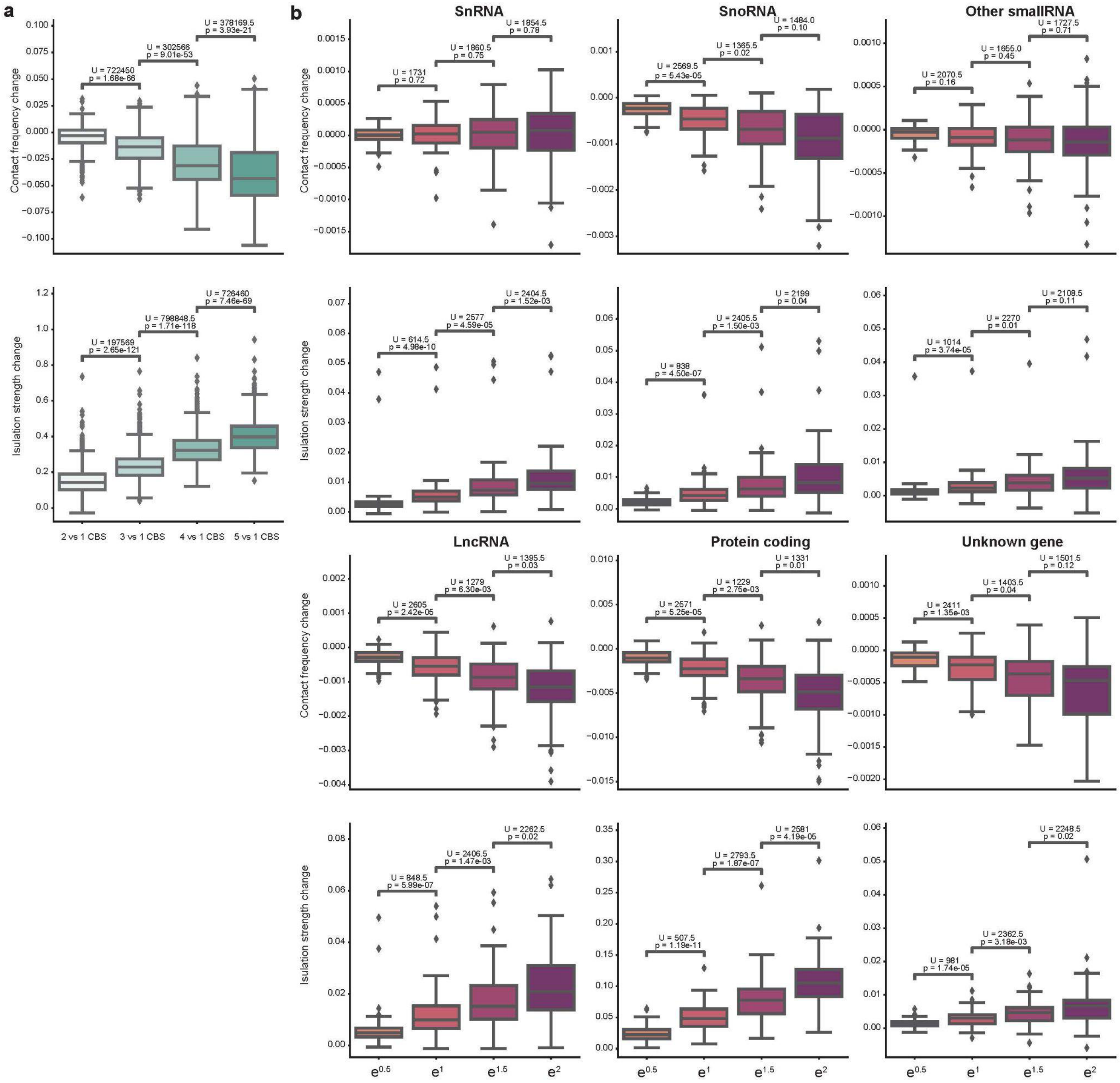

Supplementary Figure 13. *Trans*-located caRNAs might help strengthen the insulation of TAD boundaries. (a) Inserting CTCF motifs progressively from 1 to 4 to random DNA sequence (n=1,000) with a pair of convergent CTCF motifs increased the insulation strength and decreased the average contact frequency. Predictions of contact maps were made by the model with sequence alone. The center line within the box represents the median value. The box represents the interquartile ranges (IQR), with whiskers setting to 1.5 times the IQR. Outliers are shown in points. CBS: CTCF binding sites. (b) Progressively increasing the caRNA signals of each RNA type at selected TAD boundaries from 60 selected test regions by 1.6 ($e^{0.5}$) to 7.4 (e^2) fold strengthened TAD boundary insulation. Sometimes the average contact frequency of the predicted maps also decreased. Boxplots show the changes of contact frequency and insulation strength compared to the starting map without elevated caRNA signals. Predictions of contact maps were made by the model incorporating DNA sequence and *trans*-located

caRNA signals. Two-sided Mann-Whitney U tests were used to evaluate differences between each pair of comparisons. U statistics and p-values are shown in the plot. Source data are provided as a Source Data file.

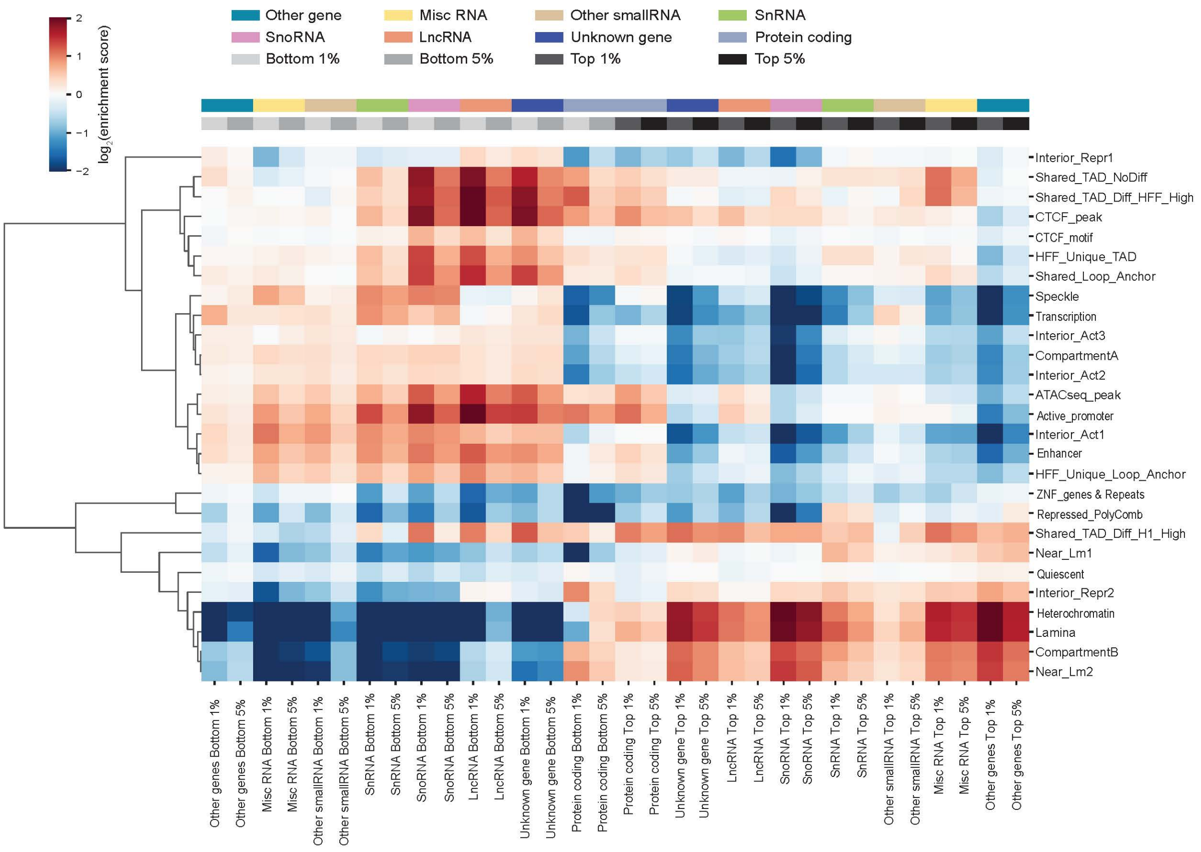

Supplementary Figure 14. Genomic regions with high absolute contribution scores from *trans*-located caRNAs show enrichment at TAD boundaries, loop anchors and nuclear structures. The heatmap shows the enrichment (log_2 enrichment score) of genomic regions with top 1%, 5% (positive) and bottom 1%, 5% (negative) contribution scores of each type of caRNAs at TAD boundaries, loop anchors, SPIN and ChromHMM states. SPIN: Spatial Position Inference of the Nuclear genome, Interior_Act 1: Interior Active 1, Interior_Act 2: Interior Active 2, Interior_Act 3: Interior Active 3, Interior_Repr1: Interior Repressive 1, Interior_Repr2: Interior Repressive 2, Near_Lm1: Near Lamina 1, Near_Lm2: Near Lamina 2. Source data are provided as a Source Data file.

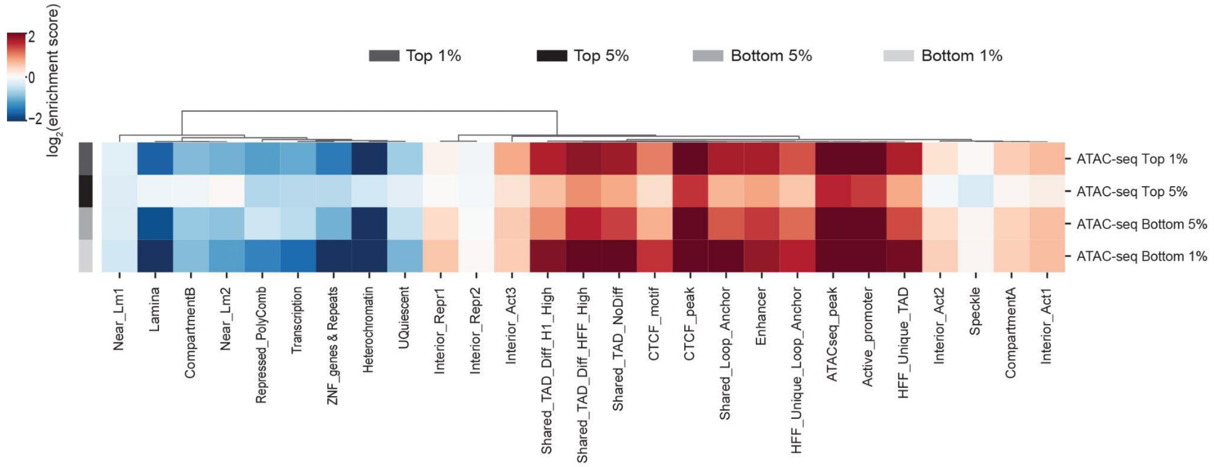

Supplementary Figure 15. Genomic regions with either top or bottom contribution scores from chromatin accessibility show enrichment in active chromatin. The heatmap shows the enrichment (log_2 enrichment score) of genomic regions with top 1%, 5% (positive) and bottom 1%, 5% (negative) contribution scores of ATAC-seq signals at TAD boundaries, loop anchors, SPIN and ChromHMM states. SPIN: Spatial Position Inference of the Nuclear genome, Interior_Act 1: Interior Active 1, Interior_Act 2: Interior Active 2, Interior_Act 3: Interior Active 3, Interior_Repr1: Interior Repressive 1, Interior_Repr2: Interior Repressive 2, Near_Lm1: Near Lamina 1, Near_Lm2: Near Lamina 2. Source data are provided as a Source Data file.

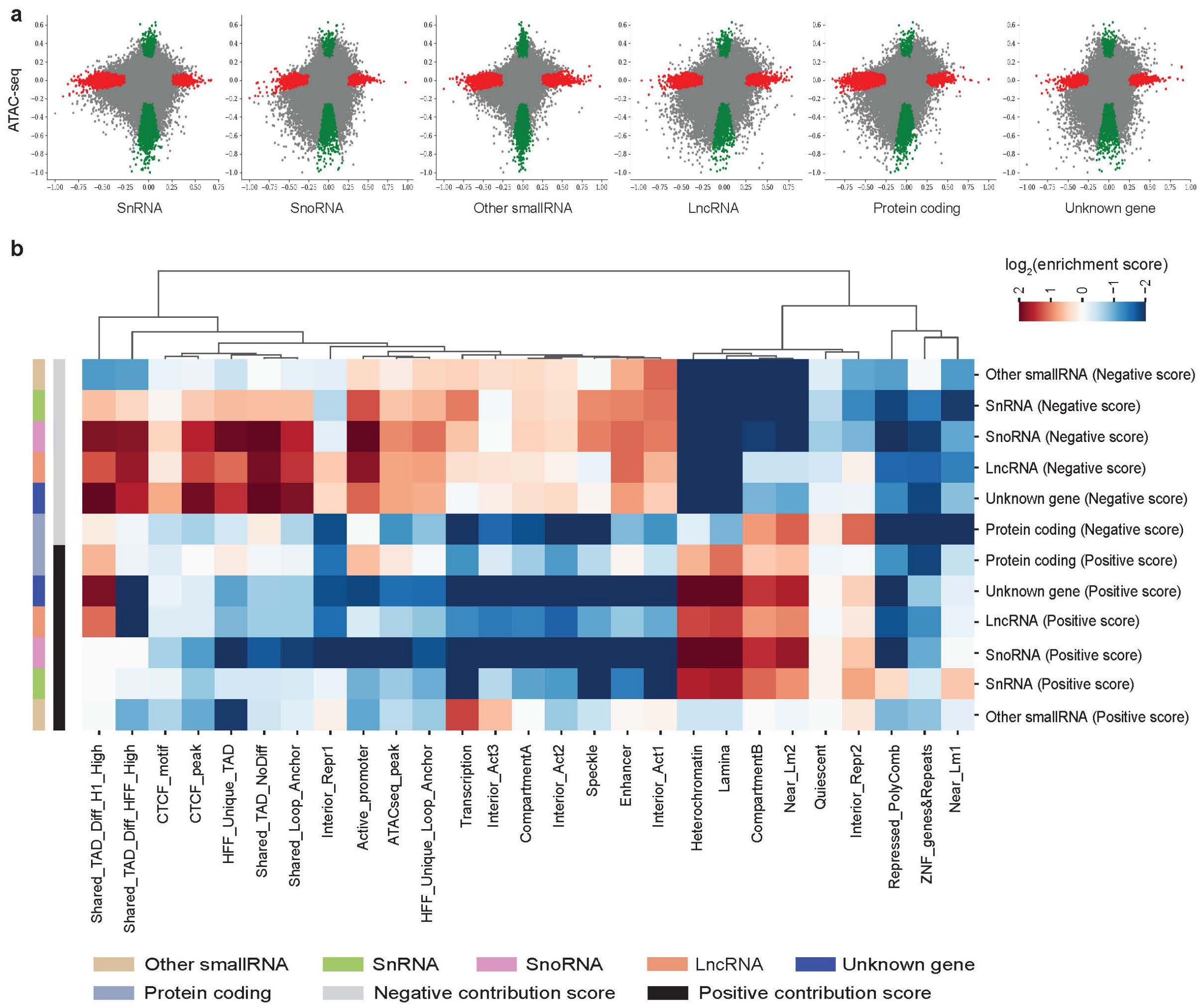

Supplementary Figure 16. Regions with higher contribution scores of *trans*-located caRNAs compared to chromatin accessibility are enriched at TAD boundaries, loop anchors and nuclear structures. (a) Comparison of contribution scores between *trans*-located caRNAs and ATAC-seq signals. (b) Enrichment ($\log_2$ enrichment score) of genomic regions that showed higher contribution scores for each type of caRNAs compared to ATAC-seq values at TAD boundaries, loop anchors, SPIN and ChromHMM states. SPIN: Spatial Position Inference of the Nuclear genome, Interior_Act 1: Interior Active 1, Interior_Act 2: Interior Active 2, Interior_Act 3: Interior Active 3, Interior_Repr1: Interior Repressive 1, Interior_Repr2: Interior Repressive 2, Near_Lm1: Near Lamina 1, Near_Lm2: Near Lamina 2. Source data are provided as a Source Data file.

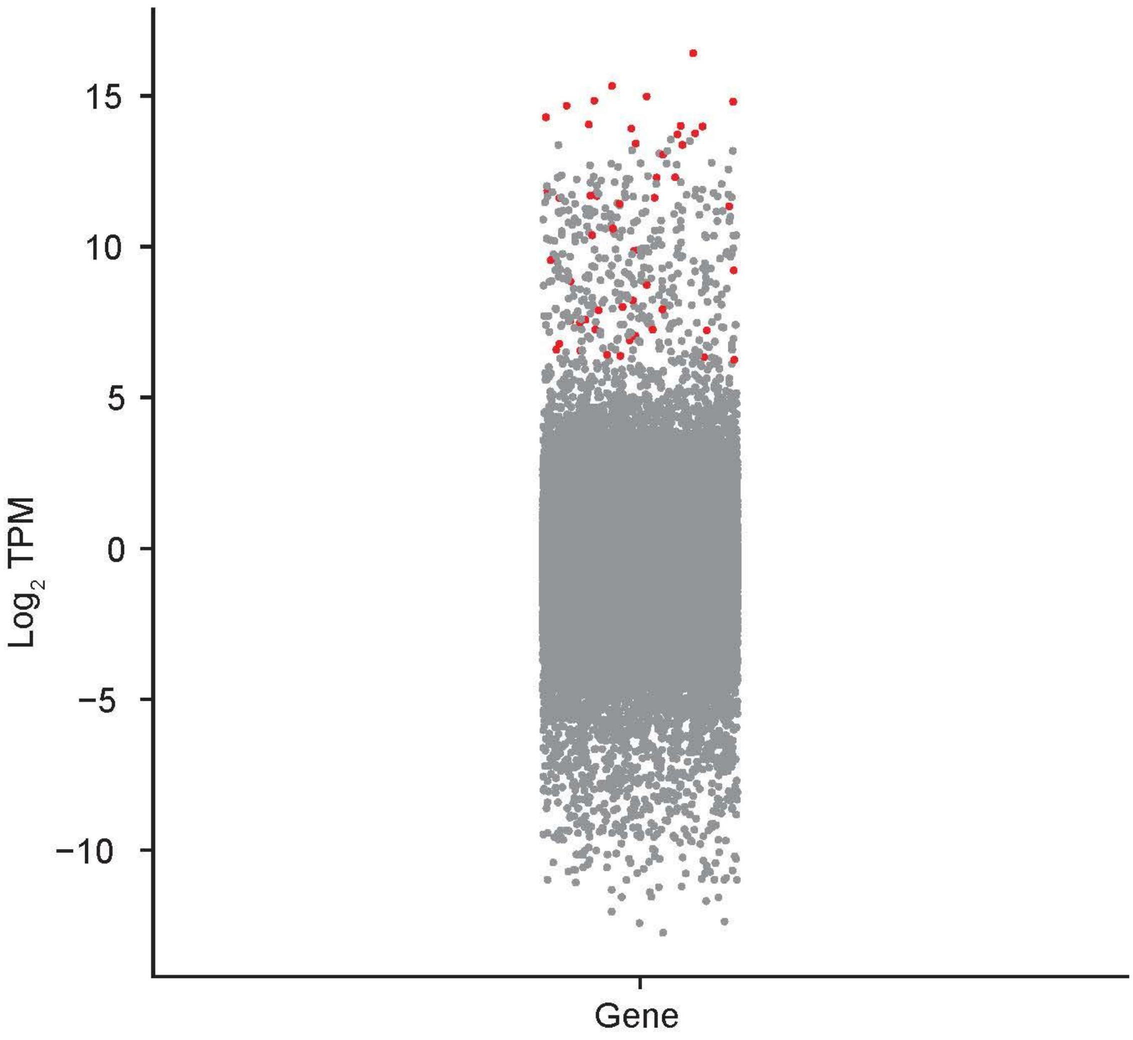

Supplementary Figure 17. Genes whose RNAs preferentially locate at genomic regions with large absolute contribution scores are highly prevalent in HFFc6. Nascent transcription (log_2TPM) of each annotated gene is shown, and the genes whose RNAs are preferentially located at genomic regions with large absolute contribution scores are highlighted in red. TPM: transcripts per million. Source data are provided as a Source Data file.

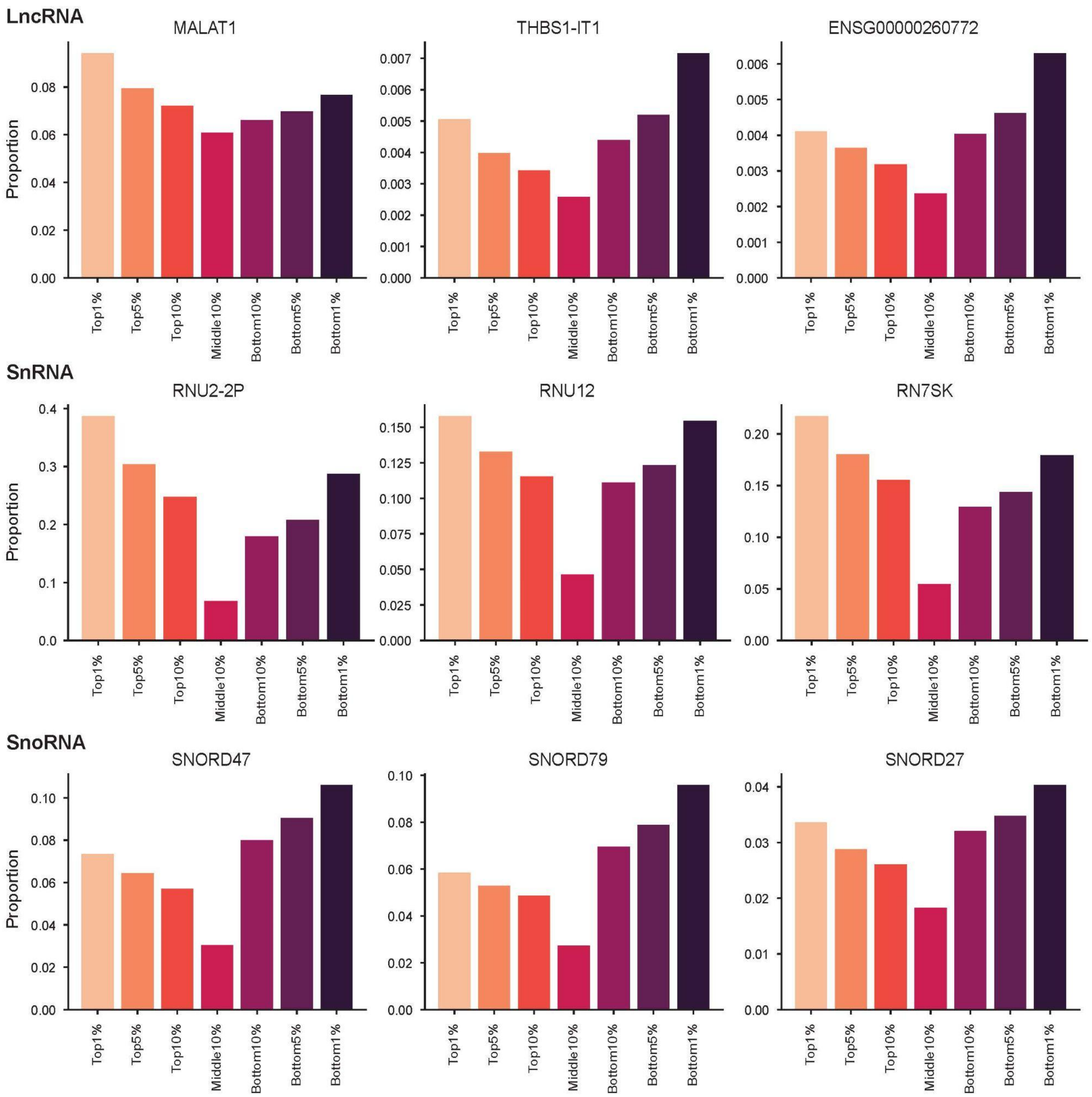

Supplementary Figure 18. Candidate RNAs that are preferentially associated with genomic regions with large absolute contribution scores. Example of lncRNAs, snRNAs and snoRNAs that preferentially interact with genomic regions with large absolute contribution scores (top 1%, top 5%, top 10%, bottom 1%, bottom 5%, bottom 10%) versus regions with lower absolute contribution scores (middle 10%). Source data are provided as a Source Data file.

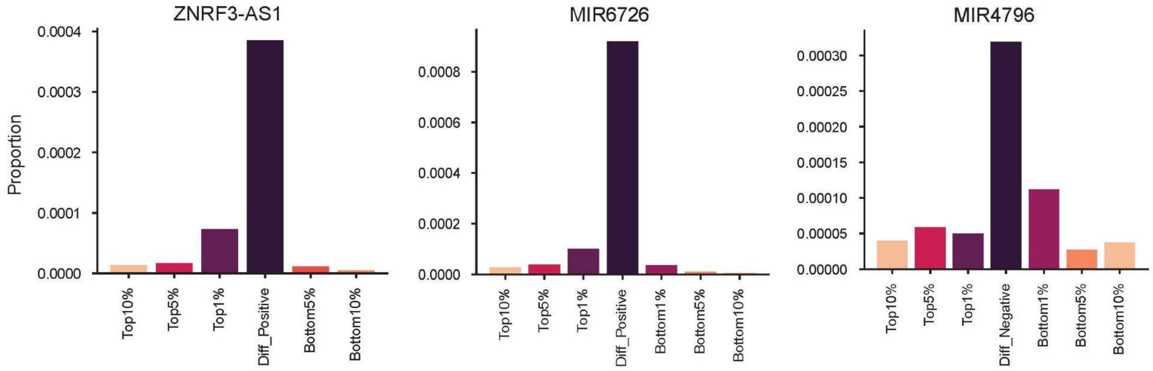

Supplementary Figure 19. Candidate RNAs that are preferentially associated with genomic regions where *trans*-located caRNAs have large absolute contribution scores and ATAC-seq features do not. Diff_Positive: differentiated regions with larger positive contribution scores for *trans*-located caRNAs. Diff_Negative: differentiated regions with larger negative contribution scores for *trans*-located caRNAs. Source data are provided as a Source Data file.
