# Supplementary material for: Exploring the roles of RNAs in chromatin architecture using deep learning
Source: Nat Commun. 2024 Jul 29;15:6373. doi: 10.1038/s41467-024-50573-w (PMC11286850; doi:10.1038/s41467-024-50573-w)
Supplement: Supplementary file 3 — Description of Additional Supplementary Information [file 41467_2024_50573_MOESM3_ESM.pdf]

Description of Additional Supplementary Files

Supplementary Data 1. Pairwise comparisons of model performance on the held-out test set regions and the cell-type-specific subsets. Two cell-type-specific subsets were selected: one identified by MSE (MSE>0.3; Cell type difference-MSE) and the other identified by MSE, SCC and SSIM (MSE>0.3, SCC<0.2 or SSIM<0.08; Cell type difference). Cell-type-specific subsets were further divided into the ones without compartment change (Same Compartment), with compartment changes from A compartment in H1ESC to B compartment in HFFc6 (AtoB), or with compartment changes from B compartment in H1ESC to A compartment in HFFc6 (BtoA). Two-sided Mann-Whitney U tests were used to evaluate differences between all pairs of models. Repeat1 and Repeat2 represents the training times of model 1 and model 2. MSE: mean squared error (MSE), SCC: stratum-adjusted correlation coefficient, SSIM: structural similarity index measure.

Supplementary Data 2. Candidate chromatin-associated RNAs that might shape chromatin architecture. Top RNAs of each type that show the most interaction with genomic regions with large absolute contribution scores (Top 5% and Bottom 5%) or the regions having higher absolute *trans*-located caRNA contribution compared to chromatin accessibility (Diff_Positive and Diff_Negative) are shown. Diff_Positive: genomic regions where *trans*-located caRNAs have large positive contribution scores and ATAC-seq features don't. Diff_Negative: genomic regions where *trans*-located caRNAs have large negative contribution scores and ATAC-seq features don't. If an RNA is one of the top 10 RNAs that show the most interaction with the abovementioned genomic regions, the genomic regions are shown in the Interacted_regions column of that RNA.

Supplementary Data 3. Candidate chromatin-associated RNAs that might shape chromatin architecture over chromatin accessibility. RNAs of each type that are preferentially associated with genomic regions where *trans*-located caRNAs have high absolute contribution scores and ATAC-seq features do not (Diff_Positive or Diff_Negative) compared to genomic regions with top 5% or bottom 5% *trans*-located caRNA contribution scores. Diff_Positive: genomic regions where *trans*-located caRNAs have large positive contribution scores and ATAC-seq features don't. Diff_Negative: genomic regions where *trans*-located

caRNAs have large negative contribution scores and ATAC-seq features don't. If an RNA displays a preference for association with Diff_Positive regions, Diff_Positive is shown in the Differentially_interacted_regions column; same for Diff_Negative.
